# A potential prognostic model based on miRNA expression profile in The Cancer Genome Atlas for bladder cancer patients

**DOI:** 10.1186/s40709-020-00116-3

**Published:** 2020-05-19

**Authors:** Yan Liu, Dong Yan Zhu, Hong Jian Xing, Yi Hou, Yan Sun

**Affiliations:** 1 Anesthesiology Department, Jilin Univ, China Japan Union Hosp, 126 Xiantai St, Changchun, 130033 Jilin People’s Republic of China; 2 Vascular Surgery Department, Jilin Univ, China Japan Union Hosp, 126 Xiantai St, Changchun, 130033 Jilin People’s Republic of China; 3 Department of Orthopedics, Jilin Univ, China Japan Union Hosp, 126 Xiantai St, Changchun, 130033 Jilin People’s Republic of China; 4 Department of Urology, Jilin Univ, China Japan Union Hosp, 126 Xiantai St, Changchun, 130033 Jilin People’s Republic of China

**Keywords:** Prognostic model, Bladder cancer, The Cancer Genome Atlas

## Abstract

**Background:**

This study aimed to construct prognostic model by screening prognostic miRNA signature of bladder cancer.

**Methods:**

The miRNA expression profile data of bladder cancer (BC) in The Cancer Genome Atlas (TCGA) were obtained and randomly divided into the training set and the validation set. Differentially expressed miRNAs (DEMs) between BC and normal control samples in the training set were firstly identified, and DEMs related to prognosis were screened by Cox Regression analysis. Then, the MiR Score system was constructed using X-Tile based cutoff points and verified in the validation set. The prognostic clinical factors are selected out by univariate and multivariate Cox Regression analysis. Finally, the mRNAs related to prognosis were screened and the biological pathway analysis was carried out.

**Results:**

We identified the 7-miRNA signature was significantly associated with the patient’s Overall Survival (OS). A prognostic model was constructed based on the prognostic 7-miRNA signature, and possessed a relative satisfying predicted ability both in the training set and validation set. In addition, univariate and multivariate Cox Regression analysis showed that age, lymphovascular invasion and MiR Score were considered as independent prognostic factors in BC patients. Furthermore, based on MiR Score prognostic model, several differentially expressed genes (DEGs), such as *WISP3* and *UNC5C*, as well as their related biological pathway(s), including cell–cell adhesion and neuroactive ligand-receptor interaction, were considered to be related to BC prognosis.

**Conclusion:**

The prognostic model which was constructed based on the prognostic 7-miRNA signature presented a high predictive ability for BC.

## Background

Bladder cancer (BC) is one of the most common malignant tumor of the urinary system, which is characterized by the high rate of non-muscle invasive BC (NMIBC) at the moment of diagnosis (75–80%) [1, 2], with approximately 3.4 million affected cases and 188,000 deaths in 2015 [3]. Currently, the standard BC screening methods include cystoscopy, sonography, and urinary cytology; however, its high invasiveness and low accuracy still cannot be neglected [4]. Although current treatments have improved 5-year survival rate of BC, most patients have delayed diagnosis of proximal and distal metastasis due to the atypical symptoms of early BC, resulting in poor treatment efficacy and prognosis. Radical cystectomy (RC) is usually performed in patients with early diagnosis of muscular invasive cystitis (MIBC). This is not the best solution because patients have a poor quality of life after surgery and a high rate of recurrence and mortality in the short time after surgery [5]. As far as patients with BC are concerned, it is impossible to predict which of them will have disease progression. Therefore, it is important to further reveal novel diagnostic and therapeutic methods, as well as underlying risk factors for poor prognosis of BC patients.

MiRNAs (miRNAs) are endogenous non-coding single-stranded small RNA molecules that regulate gene expression by repressing translational efficiency or decreasing target mRNA stability, thereby participating in various key cell biological processes, such as embryonic development, tumor cell proliferation, differentiation, and apoptosis [6, 7]. MiRNAs are known to be dysregulated in BC and implicated in the pathogenesis of bladder tumors mainly through their effect on genes involved in two molecular pathways, specifically the gene which codes tumor protein 53 (TP53) [8] and fibroblast growth factor receptor 3 (FGFR3) [9]. Previous studies have reported that the dysregulation of miRNAs is related to the prognosis and the progression of BC [1], such as miR-144-5p [10], miR-199 family [11], and miR-214 [12]. For example, Falzone et al. [13] reported that downregulated hsa-miR-145-5p and hsa-miR-214-3p may modulate the expression of both EMT and NGAL/MMP-9 pathways in BC using bioinformatic analysis. Accordingly, miRNAs may be considered good candidates as biomarkers for both prognosis and diagnosis of BC. Hence, miRNAs profiling studies from different tissues represent an excellent alternative application for these short sequences as biomarkers with clinical significance. It is widely known that poor prognosis as a major challenge for the treatment of BC leads to a low survival rate of BC patients, and gene mutation and environment exposure have been identified to be associated with this outcome. To our knowledge, however, a prognostic model of BC is rarely reported. In the current study, we aimed to develop a prognostic model for BC that provides some useful insights in improving the prognosis of BC patients and helps to increase their overall survival. For this purpose, the miRNA expression profile data of BC based on The Cancer Genome Atlas (TCGA) were analyzed to screen miRNAs related to BC prognosis and then construct a BC prognostic model using bioinformatic methods. In addition, the related clinical prognostic factors, messenger RNAs (mRNAs) and biological pathways were analyzed based on this model.

## Methods

### Data extraction and grouping

The clinical information, the miRNA expression profile and the mRNA data based on the Illumina HiSeq2000 RNA Sequencing (Illumina, San Diego, CA, USA) platform of a total of 432 samples of BC patients were downloaded from TCGA (https://gdc-portal.nci.nih.gov/) [14]. After corresponding to clinical information of them, a total of 428 samples with corresponding information were included in our study, of which 409 were tumor samples (BC group) and 19 were normal control samples (control group). Then, BC group were randomly divided into two groups: 204 tumor samples utilized as the training set and 205 tumor samples utilized as the validation set. The clinical information of tumor samples in the training set and validation set are shown in Table 1.

**Table 1:** The clinical information of bladder cancer tumor samples in the training set and validation set

Clinical characteristics	Training set (N = 204)	Validation set (N = 205)
Age (years, mean ± SD)	68.46 ± 27.57	67.69 ± 9.86
Gender (male/female)	154/50	148/57
Pathologic M (M0/M1/–)	99/7/98	96/4/105
Pathologic N (N0/N1/N2/N3/–)	122/23/36/2/21	115/23/40/6/21
Pathologic T (T1/T2/T3/T4/–)	3/65/95/27/14	1/55/99/31/19
Pathologic stage (I/II/III/IV/–)	2/71/65/65/1	0/60/74/70
Pathologic grade (high/low/–)	194/9/1	191/12/2
Radiotherapy (yes/no/–)	6/186/12	14/177/14
Lymphovascular invasion (yes/no/–)	65/73/66	85/59/61
Recurrence (yes/no)	44/126/34	43/124/38
Dead (death/alive/–)	92/110/2	86/117/2
Overall survival time (months, mean ± SD)	26.76 ± 27.57	27.45 ± 28.19

*M* distant metastases, *N* regional lymph node, *T* tumor size

### DEMs screening in the training set

First, miRNAs with median value as 0 in the training set were removed, which means the read counts obtained through the Illumina Hiseq. Next, based on the expression information provided by TCGA, limma package (version 3.34.7, https://bioconductor.org/packages/release/bioc/html/limma.html) [15] in R 3.4.1 was used to screen DEMs between BC and normal control samples with the thresholds of false discovery rate (FDR) < 0.05 and |log fold change (FC)| > 1. At last, according to the expression values of DEMs in the training set, bidirectional hierarchical clustering based on centered Pearson correlation algorithm was performed by pheatmap (version 1.0.8, https://cran.r-project.org/web/packages/pheatmap/index.html) [16] in R 3.4.1.

### DEMs screening related to prognosis

Combined with the clinical prognostic information of BC samples in the training set and the expression levels of DEMs, DEMs related to the OS were screened by Univariate Cox Regression analysis survival package (version 2.41.3, https://cran.r-project.org/web/packages/survival/index.html) [17] in R 3.4.1, with the threshold of log-rank *p*-value < 0.05.

### Construction and verification of prognosis risk assessment model based on DEMs levels

Based on DEMs related to prognosis, the optimized DEMs signature was screened using LASSO Cox Regression model [18] of penalized package (version 0.9.50, https://cran.r-project.org/web/packages/penalized/index.html) [19] in R 3.4.1. The optimized parameter lambda in this model was obtained by 1000 cycles calculation of cross-validation likelihood (cvl) algorithm. Subsequently, the cutoff value of the optimized DEM signature was calculated using X-Tile Bio-Informatics Tool (https://medicine.yale.edu/lab/rimm/research/software.aspx) [20] with the threshold of Monte-Carlo *p*-value < 0.05. The sample status was defined according to the cutoff value of each miRNA: status = 1 when the expression level of miRNA > the cutoff value; while status = 0 when the expression level of miRNA < the cutoff value [21]. After that, the risk assessment model (MiR score) was constructed for each sample by the linear combination of miRNA status weighted by regression coefficient as follows: MiR Score = ∑β_miRNA n_ × Status _miRNA n_. The β represented prognostic regression coefficient and status was defined as previously mentioned. We found that technical biases of miRNA data from the TCGA are not affecting the differential expression due to the presence of sequencing procedures or batches. According to the median value of MiR Score, all samples in the training set were divided into high risk and low risk groups. The Kaplan–Meier (K–M) survival curve analysis was used to estimate the prognosis difference between high risk and low risk groups. Meanwhile, Area Under Receiver Operating Characteristic (AUROC) curve analysis was used to assess the prognostic model. Similar to the above, all samples in the validation set were divided into high risk and low risk groups, and this model was further verified in the validation set and assessed using K–M survival analysis and Receiver Operating Characteristic (ROC) curve.

### Further analysis of the prognostic factors

The independent prognostic factors based on the clinical information of tumor samples in the training set and validation set were analyzed using univariate and multivariate Cox Regression analysis survival package (version 2.41.1) in R 3.4.1. Based on these independent prognostic factors, the nomogram of 3- and 5-year survival prediction models were constructed using rms package (version 5.1-2, https://cran.r-project.org/web/packages/rms/index.html) in R 3.4.1.

### The screening of mRNA related to prognosis and pathway analysis

The RNA-seq profile data of BC which paired with miRNA expression profile data were extracted, and then divided into high risk and low risk groups according to MiR Score. Next, differentially expressed genes (DEGs) between high risk and low risk groups were screened using limma package with the thresholds of FDR < 0.05 and log FC > 1. Following this, Gene Ontology (GO) functional annotation associated with biological process analysis [22] and Kyoto Encyclopedia of Genes and Genomes (KEGG) pathway enrichment analysis [23] were carried out using Database for Annotation, Visualization and Integrated Discovery (DAVID) program (v 6.8, https://david.ncifcrf.gov/) [24]. The *p* value < 0.05 was considered as the cutoffs for significantly statistical difference in functional analyses.

## Results

### DEMs screening in the training set

**Fig. 1:**
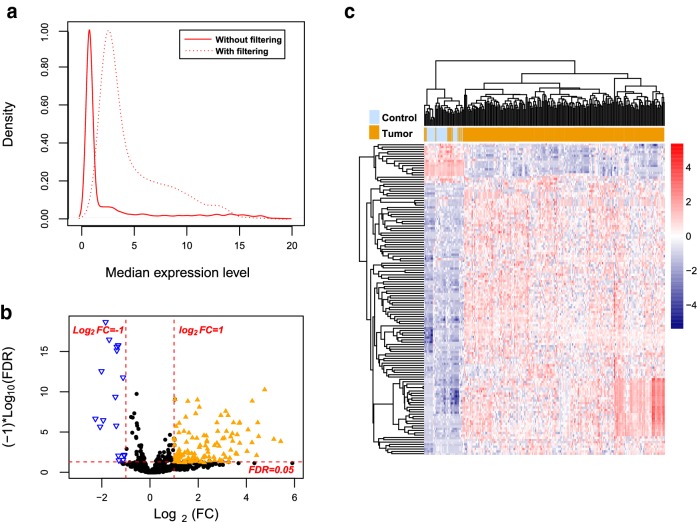
**a** The density distribution curve of miRNA expression values before and after filtering. **b** Volcano map. Orange triangle, blue triangle, and black dots indicate genes are up-regulated, down-regulated, and non-significant differentially expressed miRNAs, respectively. **c** A bidirectional hierarchical clustering map based on 134 DEGs. Blue and orange sample bars represent normal control samples and tumor samples

Among all samples contained in the training set, miRNAs with median value of read counts as 0, which indicate not expressed miRNAs, were filtered (Fig. 1a). Based on the selective criteria, a total of 134 DEMs were identified between BC and normal control samples, including 18 downregulated and 116 upregulated miRNAs (Fig. 1b). Then, bidirectional hierachical clustering was conducted for these 134 DEMs, indicating that these identified DEMs could significantly distinct tumor samples from the normal controls (Fig. 1c).

### Construction and verification of prognostic models

**Fig. 2:**
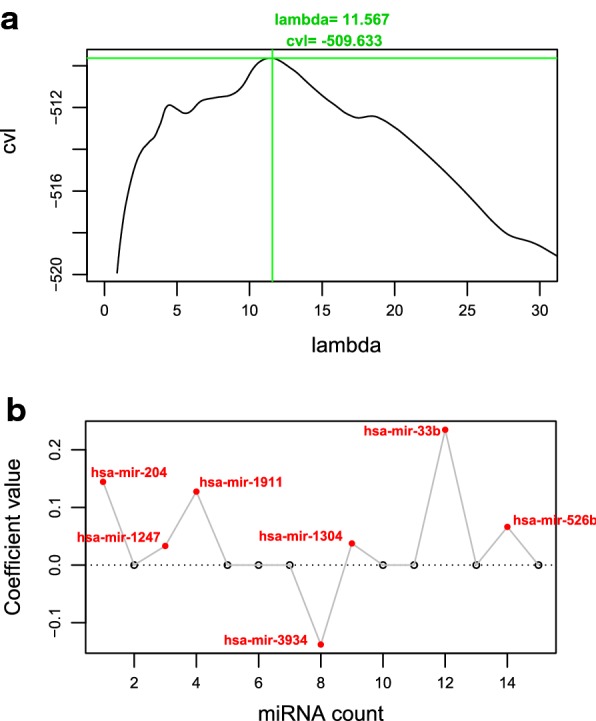
**a** The lambda parametric curves by cross-validation likelihood (cvl) algorithm. Horizontal axis and vertical axis represent lambda and cvl, respectively; the intersection of green dotted line represent that the maximum value of cvl was -509.633 when lambda was 11.567. **b** Coefficient distribution diagram of the optimized seven DEMs related to prognosis based on LASSO Cox Regression model

Among 204 BC samples in the training set, survival prognostic information was recorded in 202 BC samples. Based on the above 134 DEMs between BC and controls, univariate Cox Regression analyses were performed, and then a total of 21 DEMs were significantly associated with the patient’s OS. Based on these 21 DEMs, the optimized DEMs signatures were screened by LASSO Cox Regression model. After 1000 cycles calculation of cvl algorithm, lambda was confirmed as 11.567 and the maximum value of cvl was − 509.633 (Fig. 2a). As a result, 7 DEMs, including hsa-miR-1247, hsa-miR-1304, hsa-miR-1911, hsa-miR-204, hsa-miR-33b, hsa-miR-3934, and hsa-miR-526b, were obtained (Fig. 2b, Table 2). The cutoff value of these 7 DEMs was calculated using X-Tile Bio-Informatics Tool (Table 2).

**Table 2:** The optimized seven differentially expressed miRNAs related to prognosis in bladder cancer

miRNA_ID	β	HR	95% CI	*p*-value	X-tile cutoff
hsa-miR-1247	0.0331	1.0282	0.9199–1.1492	0.0062	2.76
hsa-miR-1304	0.0375	1.2148	0.9590–1.5388	0.0107	1.91
hsa-miR-1911	0.1274	1.2425	1.0912–1.4146	0.0010	2.42
hsa-miR-204	0.1443	1.2205	1.0321–1.4432	0.0198	0.96
hsa-miR-33b	0.2346	1.2055	0.8228–1.7662	0.0338	2.78
hsa-miR-3934	− 0.1378	0.6204	0.4611–0.8349	0.0016	1.09
hsa-miR-526b	0.0662	1.4881	0.9502–2.3303	0.0424	4.74

*β* prognostic regression coefficient; *HR* hazard ratio

**Fig. 3:**
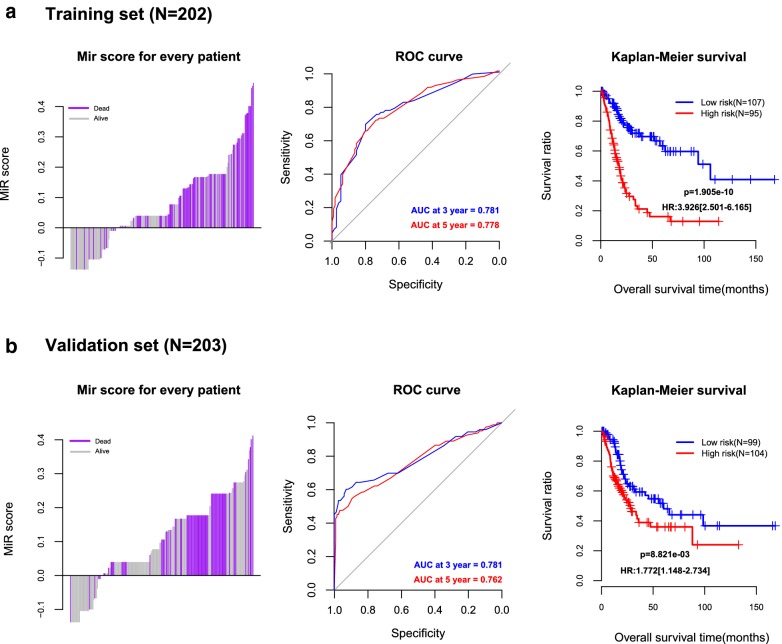
**a** The sample distribution ofthe MiR Score in the training set (left), purple and gray bars represent samples of death and survival, respectively; ROC curve analysis of 3- and 5-year survival for MiR Score risk assessment model in the training set (middle); the K–M survival analysis of low- and high- risk groups in the training set (right), HR represents hazard ratio, and the number in parentheses indicates 95% confidence interval (CI). **b** The sample distribution of the MiR Score in the validation set (left), purple and gray bars represent samples of death and survival, respectively; ROC curve analysis of 3- and 5-year survival for MiR Score risk assessment model in the validation set (middle); the K–M survival analysis of low- and high-risk groups in the validation set (right), HR represents Hazard Ratio, and the number in parentheses indicates 95% confidence interval (CI)

According to the miRNA status, the MiR Score risk assessment model was constructed as follows: MiR Score = (0.0331) × Status_hsa-mir-1247_ + (0.0375) × Status_hsa-mir-1304_ + (0.1274) × Status_hsa-mir-1911_ + (0.1443) × Status_hsa-mir-204_ + (0.2346) × Status_hsa-mir-33b_ + (− 0.1378) × Status_hsa-mir-3934_ + (0.0662) × Status_hsa-mir-526b_. The distribution of the MiR Score in the training set and validation set are shown in Fig. 3a left and b left, respectively. ROC curve analysis revealed that the area under curve (AUC) of 3- and 5-year survival were 0.781 and 0.778 in the training set, as well as 0.781 and 0.762 in the validation set, respectively, indicating that this model possessed a relative satisfying predicted ability both in the training set and validation set (Fig. 3a, b middle). Meanwhile, the estimation of K–M survival analysis showed that the OS of patients in the low-risk group was significantly longer than that in the high-risk group (*p* = 1.905 × 10^−10^, and *p* = 8.821 × 10^−3^, Fig. 3a, b right) in the training set and validation set, respectively.

### The independent prognostic factors analysis

**Fig. 4:**
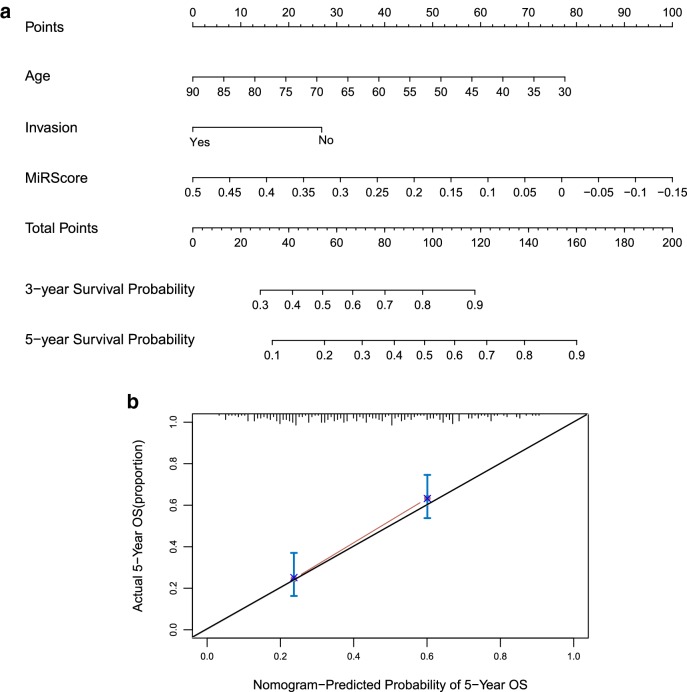
**a** The nomogram of 3- and 5-year survival prediction models for these independent prognostic factors. **b** The nomogram of 5-year survival prediction compliant to actual 5-year survival

Univariate and multivariate Cox Regression analysis showed that age > 65 years [hazard ratio = 1.016, 95% confidence interval (CI) 1.011–1.430, *p* = 2.034 × 10^−2^ and HR = 1.069, 95% CI 1.0262–1.113, *p* = 1.340 × 10^−3^], lymphovascular invasion (HR = 1.709, 95% CI 0.893–3.274, *p* = 1.050 × 10^−2^ and HR = 1.338, 95% CI 1.056–3.177, *p* = 4.509 × 10^−2^) and MiR Score (HR = 3.431, 95% CI 1.919–6.130, *p* = 3.180 × 10^−5^ and HR = 2.353, 95% CI 1.0702–5.176, *p* = 3.328 × 10^−2^) were considered as the independent prognostic factors in BC patients both in the training set and validation set, respectively (Table 3). Furthermore, the nomogram of 3- and 5-year survival prediction models of these independent prognostic factors were constructed as Fig. 4a. The nomogram of 5-year survival prediction showed compliance to actual 5-year survival (Fig. 4b).

**Table 3:** Univariate and multivariate Cox Regression analysis of tumor samples in the training set and validation set

Clinical characteristics	Uni-Cox regression		Multi-Cox regression	
	HR [95% CI]	*p*-value	HR [95% CI]	*p*-value
Training set				
Age (years, mean ± SD)	1.026 [1.006–1.047]	*9.392 × 10*^*−3*^	1.016 [1.011–1.430]	*2.034 × 10*^*−2*^
Gender (male/female)	0.996 [0.625–1.588]	9.867 × 10^−1^	–	–
Pathologic M (M0/M1/–)	1.525 [0.544–4.278]	4.180 × 10^−1^	–	–
Pathologic N (N0/N1/N2/N3/–)	1.611 [1.289–2.013]	*1.615 × 10*^*−5*^	0.999 [0.593–1.684]	6.340 × 10^−1^
Pathologic T (T1/T2/T3/T4/–)	1.594 [1.175–2.163]	*2.619 × 10*^*−3*^	0.858 [0.486–1.516]	9.980 × 10^−1^
Pathologic stage (I/II/III/IV/–)	1.661 [1.288–2.142]	*6.357 × 10*^*−5*^	1.498 [0.926–2.424]	3.970 × 10^−1^
Pathologic grade (High/low/–)	3.164 [0.439–22.77]	2.27 × 10^−1^	–	–
Radiotherapy (yes/no/–)	0.948 [0.233–3.863]	9.402 × 10^−1^	–	–
Lymphovascular invasion (yes/no/–)	2.411 [1.441–4.034]	*5.576 × 10*^*−4*^	1.709 [0.893–3.274]	*1.050 × 10*^*−2*^
Recurrence (yes/no)	1.621 [0.986–2.667]	5.473 × 10^−2^	–	–
MiR score (high/low)	3.926 [2.500–6.165]	*1.905 × 10*^*−10*^	3.431 [1.919–6.130]	*3.180 × 10*^*−5*^
Validation set				
Age (years, mean ± SD)	1.038 [1.013–1.064]	*2.272 × 10*^*−3*^	1.069 [1.0262–1.113]	*1.340 × 10*^*−3*^
Gender (male/female)	1.275 [0.808–2.011]	2.955 × 10^−1^	–	–
Pathologic M (M0/M1/–)	23.27 [6.683–81.03]	5.850 × 10^−1^	–	–
Pathologic N (N0/N1/N2/N3/–)	1.564 [1.231–1.987]	*1.749 × 10*^*−3*^	1.652 [0.854–3.195]	1.359E−01
Pathologic T (T1/T2/T3/T4/–)	1.985 [1.421–2.773]	*5.099 × 10*^*−5*^	1.858 [0.937–3.690]	7.634 × 10^−2^
Pathologic stage (I/II/III/IV/–)	1.836 [1.374–2.453]	*2.048 *× 10^−5^	0.668 [0.276–1.617]	3.710 × 10^−1^
Pathologic grade (high/low/–)	2.637 [0.365–19.07]	3.180 × 10^−1^	–	–
Radiotherapy (yes/no/–)	1.188 [0.516–2.736]	6.850 × 10^−1^	–	–
Lymphovascular invasion (yes/no/–)	2.335 [1.338–4.074]	*2.119 × 10*^*−3*^	1.338 [1.056–3.177]	*4.509 × 10*^*−2*^
Recurrence (yes/no)	2.023 [1.171–3.495]	*1.002 × 10*^*−2*^	1.002 [0.468–2.144]	9.960 × 10^−1^
MiR score (high/low)	1.772 [1.148–2.734]	*8.821 × 10*^*−3*^	2.353 [1.0702–5.176]	*3.328 × 10*^*−2*^

Italic values indicate *p* value < 0.05

*M* distant metastases, *N* regional lymph node, *T* tumor size, *HR* hazard ratio

### Functional enrichment analysis of DEGs related to prognosis

**Fig. 5:**
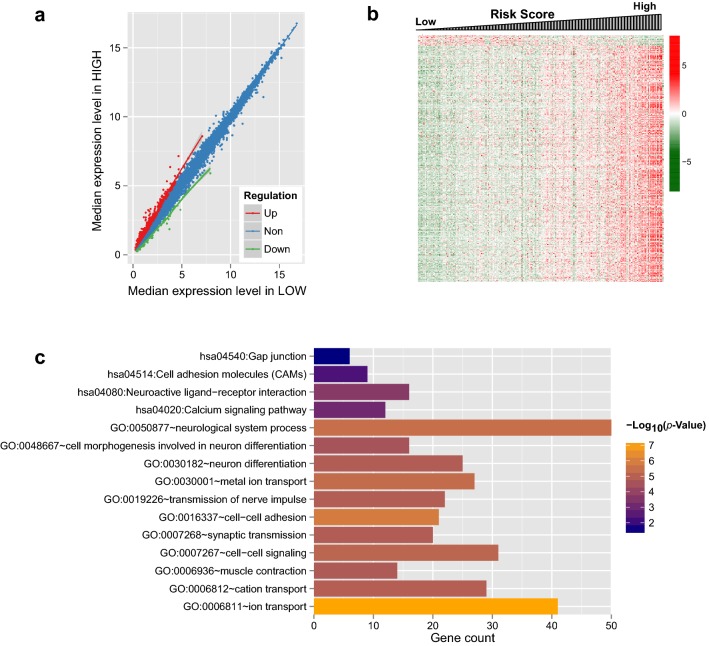
**a** Scatter plot of 389 DEGs between high risk and low risk groups. Red, green and blue dots indicate genes are up-regulated, down-regulated, and non-significant differentially expressed mRNAs, respectively. **b** Heat map of 389 DEGs between high risk and low risk groups. From left to right represents the changing process of MiRScore value from low to high. **c** GO and KEGG enrichment analyses of DEGs. Horizontal axis and vertical axis represent gene number and term, respectively; the color of the bar indicates the significant *p* value, and the closer the color is to orange, the higher the significance

Based on the selective criteria, a total of 389 DEGs were identified between high risk and low risk groups, including 33 downregulated and 356 upregulated genes, in which several DEGs, such as *WISP3* and *UNC5C* were related to biological pathway (Fig. 5a). *WISP3* and *UNC5C* are the significant DEGs between high risk and low risk group. Then, clustering analysis for these DEGs was conducted, indicating that these identified DEGs could significantly distinct high risk from low risk groups (Fig. 5b). To further identify the functional characteristics of DEGs, the functional enrichment analyses of genes were conducted with DAVID. Consequently, the Biology Process (BP) analysis of DEGs revealed that the significant enriched terms primarily concentrated on ion transport, cell–cell adhesion, neurological system process, metal ion transport, cell–cell signaling, cation transport, transmission of nerve impulse, neuron differentiation, synaptic transmission, muscle contraction, and cell morphogenesis involved in neuron differentiation (Fig. 5c). In addition, the KEGG pathway analysis implied that these DEGs were responsible for neuroactive ligand-receptor interaction, calcium signaling pathway, cell adhesion molecules, and gap junction pathways (Fig. 5c, Table 4).

**Table 4:** GO and KEGG enrichment analyses of DEGs

Category	Term	Count	*p*-value	Genes
Biology process	GO:0006811 ~ ion transport	41	5.51 × 10^−8^	SLC8A3, SLC36A2, GABRB3
	GO:0016337 ~ cell–cell adhesion	21	1.15 × 10^−6^	HCN4, SLC4A4, CAMK2A,
	GO:0050877 ~ neurological system process	50	3.23 × 10^−6^	IGDCC3, OR10A3, GABRB3
	GO:0030001 ~ metal ion transport	27	3.97 × 10^−6^	CPLX3, DRD2, OPRK1
	GO:0007267 ~ cell–cell signaling	31	6.68 × 10^−6^	LIN7A, SFRP5, PENK
	GO:0006812 ~ cation transport	29	1.09 × 10^−5^	SLC8A3, SLC36A2, DRD2
	GO:0019226 ~ transmission of nerve impulse	22	1.21 × 10^−5^	SLC24A5, CACNG4, KCNK2
	GO:0030182 ~ neuron differentiation	25	1.34 × 10^−5^	TUBB2B, RTN4RL1, PTPRZ1
	GO:0007268 ~ synaptic transmission	20	1.37 × 10^−5^	CPLX3, PLP1
	GO:0006936 ~ muscle contraction	14	1.69 × 10^−5^	ACTC1, MYBPC2, DRD2
	GO:0048667 ~ cell morphogenesis involved in neuron differentiation	16	2.87 × 10^−5^	RTN4RL1, DRD2, PTPRZ1
KEGG pathway	hsa04080:Neuroactive ligand-receptor interaction	16	2.10 × 10^−4^	GPR156, CALCR, GABRB3
	hsa04020:Calcium signaling pathway	12	9.18 × 10^−4^	SLC8A3, ATP2B2, ADRB3
	hsa04514:Cell adhesion molecules (CAMs)	9	0.00562107	NCAM1, NCAM2, CADM3
	hsa04540:Gap junction	6	0.036085973	ADCY2, TUBB2B, DRD2

## Discussion

In this study, 21 miRNAs related to BC prognosis were identified based on the expression levels in TCGA by univariate and multivariate Cox Regression analysis. Then, seven out of them were further isolated as the optimized prognostic gene signature and a MiR Score prognostic model was constructed based on these seven miRNAs (hsa-mir-1247, hsa-mir-1304, hsa-mir-1911, hsa-mir-204, hsa-mir-33b, hsa-mir-3934 and hsa-mir-526b), which presented a relative highly forecast ability for BC. In addition, age, lymphovascular invasion and MiR Score were identified as the independent prognostic factors in BC patients from TCGA. Furthermore, based on MiR Score prognostic model, several DEGs, such as *WISP3* and *UNC5C*, as well as their related pathway, including cell–cell adhesion and neuroactive ligand-receptor interaction, were considered to be related to BC prognosis. Besides, we found *UNC5C* is a potential target for hsa-mir-1911, hsa-mir-3934 and hsa-mir-526b through Targetscan, which belongs to the UNC-5 family of netrin receptors. Netrins are secreted proteins that direct axon extension and cell migration during neural development (NCBI, https://www.ncbi.nlm.nih.gov/). At present, studies have indicated that *UNC5C* was associated with colorectal cancer [25, 26] and Alzheimer’s Disease [27]. Thus, further studies are needed to identify the association between *UNC5C* and hsa-mir-1911, hsa-mir-3934 and hsa-mir-526b in BC patients.

The mining of a large amount of genetic data in various diseases have been enhanced due to the rapid technological advances in high-throughput sequencing and bioinformatics [28]. TCGA, as a public and available cancer genomic database, provides comprehensive data for different types of cancer, including mRNA expression data, miRNA expression data, copy number variation, DNA methylation, and clinical information [29]. The data from TCGA have been effectively applied to improve diagnostic and therapeutic methods of cancers, as well as finally cancer prevention [29]. Thus, this study was also performed based on the miRNA expression profile data and clinical information of BC form TCGA. MiRNA expression profiles have been reported to predict the prognosis outcome of cancers [30]. Computationally, univariate and multivariate Cox Regression were the most common method to construct the prognostic models and screen prognostic factors [31]. In this study, the Cox Regression model based on the LASSO, a semi-parametric proportional hazards model, was applied. The availability of this model in survival analysis have been confirmed in recent studies [32, 33]. Similarly, in this study, the MiR Score prognostic model constructed by LASSO Cox Regression model showed a higher predictive ability both in training and validation sets. In addition, this study showed that age and lymphovascular invasion were independent prognostic factors in BC patients. Consistent with our results, previous studies have also demonstrated that age and lymphovascular invasion are associated with poor prognosis in BC patients [34], Notably, MiR Score was also been considered as an independent prognostic factor in BC patients, which further showed that the MiR Score prognostic model had a significant predictive ability for BC prognosis.

In this study, the 7-miRNA signature was identified. Specifically, miR-1247, as a tumor suppressor, has been reported in several cancers, including lung cancer [35], hepatocellular cancer [36], and pancreatic cancer [37]. A recent study also has shown that miR-1247 inhibits cell proliferation and invasion through down-regulating its target gene *RAB36* in BC [38]. MiR-204, miR-33b, and miR-526b are three reported miRNAs that function as tumor suppressor in cancers. Previous studies have suggested that miR-204 plays an inhibitory effect on cell invasion in gastric cancer cells [39] and non-small cell lung cancer [40]. MiR-33b has been reported to inhibit migration and invasion in osteosarcoma cells [41], melanoma cells [42], and lung adenocarcinoma cells [43]. In addition, miR-526b is revealed to have an inhibitory role in non-small cell lung cancer [44]. However, studies of these three miRNAs as well as miR-1304, miR-1911, and miR-3934 are rarely reported in BC. Therefore, it is important to further reveal the mechanism and prognostic significance of 7-miRNA signature in BC.

Furthermore, this study found that several DEGs, such as *WISP3* and *UNC5C*, were closely associated with BC prognosis. *WISP3*, also known as cellular communication network factor 6 (CCN6), is a cysteine-rich and glycosylated signaling protein and key regulatory extracellular matrix component [45]. It is one member of the CCN (Cyr61, CTGF, Nov) family which play important roles in several biological functions, including cell proliferation, adhesion, and invasion [45], which suggested the close relationship of *WISP3* and cell–cell adhesion. Increasing evidences have demonstrated that *WISP3* is abnormally expressed in cancers and play a contrary effect in cancer progression [46]. It is reported that *WISP3* is overexpressed as an oncogene in ovarian carcinomas [47]. On the contrary, *WISP3* is a tumor suppressor gene that inhibits cell proliferation in breast cancer [48]. Zeng et al. [49] has found that depletion of *WISP3* notably inhibited the invasion of BC cells. Our data suggests that inhibition of *WISP3* may be a therapeutic strategy for BC. Recent study has found that *WISP3* is up-regulated and promotes the cell proliferation and invasion in BC cells, which is consistent with our results. *UNC5C* (unc-5 netrin receptor C) belongs to the netrin-1 receptor family, and plays key role in cell apoptotic process as a dependence receptor that may be involved in neuroactive ligand-receptor interaction [50]. Previous reports have suggested that *UNC5C* has tumor suppressive effect in colorectal cancer through promoter methylation [51]. In addition, *UNC5C* is reported to be downregulated due to specific genetic alterations and inhibits apoptosis of tumor cells by suppressing proapoptotic signals [52]. More recently, the receptors of *UNC5* family have been revealed to be involved in the regulation of cell death processes in BC [53, 54]. However, there were still a number of limitations in the work. Functional validation was lacked for the feature genes obtained herein. Further investigations for these genes are required with substantial experiments. Nevertheless, this work provides novel insight into the pathogenesis of BC.

## Conclusion

In conclusion, the prognostic model based on the prognostic 7-miRNA signature presented a relatively promising predictive ability for BC. The seven prognostic miRNAs may have clinical implications in BC prognosis. However, the prognostic significance of 7-miRNA signature in BC should be futher confirmed in clinical study.

## Data Availability

The raw data were collected and analyzed by the Authors, and are not ready to share their data because the data have not been published.

## Abbreviations

BC: Bladder cancer
TCGA: The Cancer Genome Atlas
DEGs: Differentially expressed Genes
DEMs: Differentially expressed miRNAs
OS: Overall survival
miRNA: microRNA
FDR: False discovery rate
FC: Fold change
MiR Score: miRNA prognosis score
KEGG: Kyoto Encyclopedia of Genes and Genomes
